# TGase positively regulates photosynthesis via activation of Calvin cycle enzymes in tomato

**DOI:** 10.1038/s41438-019-0173-z

**Published:** 2019-08-01

**Authors:** Min Zhong, Yu Wang, Kun Hou, Sheng Shu, Jin Sun, Shirong Guo

**Affiliations:** 1 Key Laboratory of Southern Vegetable Crop Genetic Improvement, Ministry of Agriculture, College of Horticulture, Nanjing Agricultural University, 210095 Nanjing, China; 2 Suqian Academy of Protected Horticulture, Nanjing Agricultural University, 223800 Suqian, China

**Keywords:** Photosynthesis, Plant physiology

## Abstract

Transglutaminases (TGases), which are widespread cross-linking enzymes in plants, play key roles in photosynthesis and abiotic/biotic stress responses; however, evidence concerning the genetics underlying how TGase improves the capability of photosynthesis and the mechanism of TGase-mediated photosynthesis are not clear in this crop species. In this study, we clarified the function of TGase in the regulation of photosynthesis in tomato by comparing wild-type (WT) plants, *tgase* mutants generated by the CRISPR/Cas9 system and *TGase*-overexpressing (*TGase*OE) plants. Our results showed that increasing the transcript level of *TGase* resulted in an enhanced net photosynthetic rate (Pn), whereas the *tgase* mutants presented significantly inhibited Pns and CO_2_ assimilation compared with the WT. Although the total RuBisCO activity was not affected by TGase, the initial and activation status of RuBisCO and the activity of RuBisCO activase (RCA) and fructose-1,6-bisphosphatase (FBPase) in *TGase*OE plants were significantly higher than that in WT plants. Except for RuBisCO small subunit (RbcS), the transcription levels of Benson–Calvin cycle-related genes were positively related to the endogenous TGase activity. Furthermore, *TGase*OE plants had higher protein levels of RuBisCO large subunit (RbcL) and RCA than did WT plants and showed a reduced redox status by enhancing the activity of dehydroascorbate reductase (DHAR) and glutathione reductase (GR), which was compromised in *TGase*-deficient plants. Overall, TGase positively regulated photosynthesis by maintaining the activation states of the Benson–Calvin cycle and inducing changes in cellular redox homeostasis in tomato.

## Introduction

It is well known that photosynthesis is considered the most important metabolic process for biomass accumulation in plants but is limited by carbohydrates and various environmental cues. Therefore, understanding the mechanisms of regulation of photosynthetic processes and improving crop yield potential are critical. Interestingly, increasing evidence supports the hypothesis that polyamines (PAs) regulate photosynthetic capability^1,2^. It has been reported that PAs are usually found in the photosynthetic apparatus and are closely related to plant growth and stress responses^3^. Overexpressing the genes involved in PA biosynthesis results in increased photosynthetic rates (Pns) in transgenic plants^4,5^. In addition, putrescine (Put) plays a positive role in stimulating photophosphorylation, at least to some degree, and regulates adenosine triphosphate synthesis^6,7^. PAs are an important regulator of redox homeostasis, which increase photosynthesis because of the activation of antioxidant enzymes and the modulation of reactive oxygen species (ROS) homeostasis^8,9^. However, the functions of PA-regulated photosynthesis are not fully understood.

The regulation of PAs is often mediated by transglutaminases (TGases), which are able to establish ε-(γ-glutamyl) links with PAs for the posttranslational modification of proteins. For instance, PAs cross-linked with tubulin and actin via TGase have been observed during the germination of *Malus domestica* pollen^10,11^. TGases are intracellular and extracellular enzymes that are actively regulated by Ca^2+^ and can be negatively regulated by guanosine triphosphate. It has been demonstrated that TGases exhibit a large number of functions both in eukaryotes and prokaryotes^12^. In addition, a large amount of TGases are present in the mitochondria, cell walls, and chloroplasts of plants and are continually being identified^13–16^. In addition, the first TGase gene discovered was *AtPng1p*, which is most studied gene encoding a TGase protein in *Arabidopsis*; *AtPng1p* is expressed under conditions of undisturbed growth and expressed ubiquitously during all stages of plant growth and can be induced by various light conditions^14,17^. Furthermore, TGases are delivered via a membrane/cytoskeleton-based transport system to the cell wall, where they regulate the apical growth of pollen tubes^18^. Numerous reports have shown that TGase plays roles not only in nonphotosynthetic tissues but also in photosynthetic organs. Recent proteomic and transcriptomic studies have shown that chloroplast proteins (i.e., light-harvesting complexes of photosystem II (LHCII) and chlorophyll-binding protein of 29, 26, and 24 kDa) are the target proteins of TGase in vivo^19^. Chloroplast-associated proteins such as the larger subunit of RuBisCO (RbcL) and photosystem II proteins are substrates of TGase in higher plants^16,20^. In this respect, TGase may have positive roles in photosynthesis or photoprotection reactions. Characterization of transgenic tobacco overexpressing TGase revealed that TGase has a positive role that involves the photosystems^21^. In addition, photosynthetic complexes are often significantly affected by TGase^22^. However, the role of TGase in photosynthesis is still unclear. Researchers have been focused on the posttranslational modification of LHCII and the polyamination of thylakoids by TGase, but the function of TGase in redox regulation is often overlooked. PAs may be donors for the biosynthesis of amino acids such as glutamic acid, a key chlorophyll and glutathione precursor; in addition, PAs can regulate plastidial membrane assembly or take part in redox regulation, which has been indicated to be mediated by TGase^23^. Moreover, some TGases have been identified in rice and maize, but their functions and regulatory mechanisms are largely unknown, especially in horticultural plant species.

Tomato is one of the most economically important horticultural crop species and is distributed worldwide, and this species been widely used to study photosynthesis. The clustered regulatory interspaced short palindromic repeats/CRISPR-associated protein 9 (CRISPR/Cas9) gene editing technology has been established and used to characterize gene functions in plants^24^. In this study, to analyze the potential role of *TGase* in tomato, we generated *TGase*-overexpressing (*TGase*OE) plants and *tgase* mutants by CRISPR/Cas9 technology and compared their photosynthetic capability with that of wild-type (WT) plants. We found a positive relationship between endogenous TGase activity and the capability of photosynthesis. TGase improved photosynthetic capability by altering the cellular redox status and activating the antioxidant capability.

## Materials and methods

### Plant materials and growth conditions

Tomato (*Solanum lycopersicum* L. cv Ailsa Craig) was used for expression analysis. The seedlings were grown in a greenhouse at Nanjing Agriculture University in Nanjing city under standard water management and pest control regimens.

### Generation and selection of transgenic plants

To generate *TGase*OE plants, the CDS of *TGase* was obtained via PCR amplification using the primers *TGase*OE-F (5′-TTGGCGCGCCATGGTTGCTCGGAGACTCGCCGTTA-3′) and *TGase*OE-R (5′-CGGGGTACCACTGCTACCTGCAAAGAGGTCAATG-3′) according to the sequence (Sol Genomic Network accession Solyc01g097440). The PCR product was inserted into the binary plasmid vector pFGC1008-HA with a 35S promoter after being digested with *Asc*I and *Kpn*I. The vector was then transformed into *Agrobacterium tumefaciens* strain EHA105, which was then inserted into Ailsa Craig plants following the method of Fillatti et al^25^. The overexpression of *TGase* in plants was identified by real-time quantitative PCR (qPCR) analysis (Fig. S1). Two homozygous F2 lines (OE-#1 and OE-#2) were chosen for the experiment.

To obtain mutants, we used the CRISPR/Cas9 genome-targeting system to modify the *TGase* according to previous methods^26^. In the *TGase* coding region, we chose the guide RNA sequence GGCCCTTCAGTCTCATTACC as the single guide RNA (sgRNA). Double-stranded DNA, generated by annealing the oligo pairs, was inserted into an AtU6-sgRNA-AtUBQ-Cas9 vector, which was then digested by *Hind*III and *Kpn*I and subsequently introduced into a pCAMBIA1301 vector using T4 DNA ligase (Thermo Fisher Scientific, Waltham, USA, EL0013). The integrated constructs were subsequently transformed into EHA105 bacteria, which were then inserted into Ailsa Craig plants; hygromycin was then used to screen the transgenic plants. The genomic DNA of the mutant seedlings was extracted for PCR using specific primers (Table S1). The PCR products of the mutants were detected by direct sequencing methods. The *tgase-1* and *tgase-2* mutants, which carried a 10 bp deletion and a 1 bp addition, respectively, were identified and used in this study (Fig. S2).

In the experiments, five genotypes, Ailsa Craig (WT) plants, *TGase*-deficient mutants (*tgase-1* and *tgase-2*), and overexpression plants (OE-#1 and OE-#2), were used. To analyze photosynthetic capability, germinated seeds were sown in growth media. After full development of the second true leaves, the seedlings were transplanted into 250 cm^3^ plastic pots that contained media and were watered with 1/2-strength Hoagland’s nutrition solution. Forty-five-day-old plants were used for the experiment. For assay the Calvin cycle enzyme activity, leaf discs were harvested from different genotypes, then frozen immediately in liquid nitrogen, and stored at −80 °C prior to analysis. For detection the activity of TGase and antioxidant enzymes, the leaf samples were collected based on fresh weight. For analysis the gene expression, the whole leaflets were collected.

### TGase activity analysis

The hydroxamate method was used to calculate the transglutaminase activity, with some modifications^27^. Leaf samples (0.3 g) were homogenized with phosphate buffer (50 mM, pH 7.8) at 12,000 g for 15 min at 4 °C for endogenous TGase activity measurement. The colorimetric method (450 nm) was used to measure the activity of TGase in conjunction with N-carbobenzoxy-l-glutaminylglycin (CBZ–Cln–Gly–OH), which is the specific substrate of TGase. The 100 μl of the supernatant was fully mixed with the reaction (50 mM PBS, and 0.5 mM CBZ–Cln–Gly–OH, pH 7.8), then performed at 30 °C for 10 min and performed with 5 mM Ca^2+^; replaced by 1 mM EDTA in the negative control. l-glutamic acid γ-mono-hydroxamate was used to prepare the standard curve. The enzyme activity was defined as the amount of enzyme that catalyzed the formation of 1μmol of hydroxamate/min.

### Leaf gas exchange measurements

Forty-five-day-old plants were used for comparing photosynthetic capabilities. Gas exchange analysis was performed via an LI-6400 portable photosynthesis system (LI-6400; LI-COR, Lincoln, NE, USA). An assimilation vs intercellular CO_2_ concentration (A/Ci) curve was generated as described by Caemmerer and Farquhar^28^. The maximum rates of Rubisco (*V*_c,max_) and the maximum rate of electron transport for RuBP regeneration (*J*_max_) were measured according to the A/Ci curves as described by Ethier and Livingston^29^.

### Determination of Rubisco, Rubisco activase (RCA), and fructose-1, 6-bisphosphatase (FBPase) activity

The Rubisco activity was measured following the method of Ward and Keys^30^, with slight modifications. Leaf samples were ground with extraction buffer (2% insoluble PVPP, 1 mM EDTA, 50 mM HEPES, 10 mM MgCl_2_, and 10 mM β-mercaptoethanol; pH 8.0) and then centrifuged at 12,000 *g* for 15 min at 4 °C. The crude extract was used to assay the total activity. Activation was performed in a 100 μl mixture solution at 28 °C for 15 min. Initial Rubisco activity was determined in a 100 μl reaction medium, and the change in absorbance within 90 s was measured at 340 nm. The RCA activity was measured with a Rubisco Activase Assay Kit (Genmed Scientifics, Wilmington, USA) according to the manufacturer’s instructions.

The activity of FBPase was detected by measuring the increase at *A*_340nm_ as previously described^31^. The total activity of FBPase was determined using the crude extract, which was activated in a 100 μl activation mixture reaction (0.1 M Tris-HCl (pH 8.0), 2 mM fructose-1,6-bisphosphate (FBP), 100 mM dithiothreitol (DTT), and 10 mM MgCl_2_). After homogenization, the initial activity was measured immediately. The reaction was started by the addition of the enzyme extract.

### Glutathione content and ascorbate content assays

To determine the glutathione and ascorbate contents, leaf tissue was ground in 2 ml of extraction buffer (6% metaphosphoric acid) and then centrifuged at 12,000 *g* for 10 min at 4 °C, after which the supernatant was used for further analysis. To assay the GSH and oxidized glutathione (GSSG), the neutralized supernatant with phosphate buffer (0.5 M, pH 7.5) was added to the reaction mixture (100 mM phosphate buffer, 5 mM EDTA, 0.2 mM NADPH, and 0.6 mM 5,5′-dithio-bis (2-nitrobenzoic acid), pH 7.5). After adding the glutathione reductase (GR) (Sigma, Santa Clara, CA, USA) to start the reaction, the changes in absorbance within 1 min at 412 nm were measured. The GSH was masked after adding 40 μl of 2-vinylpyridine to the supernatant for the GSSG assay and the total glutathione assay by adding 40 μl of water. The GSH content was measured by removing the GSSG content from the total content^32^.

To assay the total levels of AsA, 20 μl of the supernatant was transferred to a new tube containing potassium phosphate buffer (0.5 M, pH 7.4); the same amount of 5 mM DTT was added as was the supernatant, after which the mixture was incubated at 37 °C for 20 min. Afterward, 10 μl of *N*-ethylmaleimide (NEM, 0.5% w/v) was added to clear the excess DTT via incubation for 1 min at 25 °C. A color reagent (80 μl) (see below) was added to the mixture, which was then incubated at 37 °C for 1 h; the absorbance was measured at 550 nm. To analyze the reduced AsA, 20 μl of potassium phosphate (0.4 M, pH 7.4) was replaced with DTT and NEM, and the procedure was performed according to the total AsA assay. The color reagent was as follows: solution A contained 31% orthophosphoric acid, 0.6% (w/v) iron chloride (FeCl_3_), and 4.6% (w/v) TCA, whereas solution B contained 4% 2,2-dipyridyl.

### Measurements of the activity related to AsA–GSH cycle enzymes

To analyze the enzymatic activities related to the AsA–GSH cycle, leaf samples were ground in 3 ml of extraction buffer (25 mM HEPES, 2% PVP, 2 mM ascorbic acid, and 0.2 mM EDTA, pH 7.8) and centrifuged at 12,000 *g* for 20 min at 4 °C. The supernatants were then collected and used for the enzymatic activity assay. The activities of ascorbate peroxidase (APX) and dehydroascorbate reductase (DHAR) were evaluated by monitoring the decrease and increase in absorbance at 290 nm and 265 nm, respectively, as described by Nakano and Asada^33^. To analyze the activity of GR, we monitored the rate of NADPH decrease according to the method reported by Halliwell and Foyer^34^.

### RNA isolation and reverse-transcription qPCR

Tomato leaves were used to extract total RNA following the manufacturer’s instructions of an RNA extraction kit (Tiangen, Beijing, China). Total RNA (1 μg) was used to synthesize cDNA using a HiScript™ Q RT SuperMix for qPCR (+gDNA wiper) kit (Vazyme, Nanjing, China). qPCR analyses were performed using an ABI ViiA7 real-time PCR system (Applied Biosystems, CA, USA). The PCR program consisted of predenaturation at 95 °C for 5 min, followed by 40 cycles of 95 °C for 10 s and 60 °C for 30 s. The *Actin* gene was used as a reference gene, and the qPCR-specific primers are presented in supplementary Table S1. The relative levels for each expression were calculated according to the methods of Livak and Schmittgen^35^.

### Western blot analysis

Proteins were extracted as described previously^35^. The proteins were resolved using 12% (w/v) SDS–polyacrylamide gel electrophoresis and then transferred to a nitrocellulose membrane (Millipore, Saint-Quentin, France)^36^. RuBisCO large subunit (RbcL), RuBisCO small subunit (RbcS), and RCA were detected with commercial antibodies (Agrisera, Vannes, Sweden). At least three independent replicates were used for each determination. The accumulation of proteins was quantified using Quantity One software (Bio-rad, Hercules, California, CA, USA).

### Statistics

The data are presented as the means ± SDs and were analyzed by SPSS 20 statistical software. The experimental data were analyzed with Duncan’s multiple range test at *P* < 0.05.

## Results

### Expression pattern of *TGase* in tomato

To detect the expression pattern of *TGase*, the mRNA transcripts of *TGase* were measured in different tissues, including the roots, stems, leaves, flowers, and fruits, in tomato (Fig. S1). Transcripts of *TGase* were highly expressed in the leaves and fruits and accumulated in the flowers (Fig. S1).

To explore the functions of TGase, we first generated *TGase*OE tomato lines and obtained several independent transgenic lines. Moreover, the transcripts of *TGase* in two overexpression lines, OE-#1 and OE-#2, showed 117.5 and 124.3 times greater expression than the WT plants did, respectively (Fig. S2). We also used the CRISPR/Cas9 system to generate mutants, which were named *tgase-1* and *tgase-2*, with a loss of 10 bp and an “A” insertion in an exon of *TGase*, respectively (Fig. S3). In the mutants, the activity of TGase was almost undetectable; in contrast, the activity of TGase increased in the *TGase*OE plants (Fig. S4). These results indicated that *TGase* deficiency significantly inhibited TGase activity but dramatically increased *TGase*OE activity. These homozygous overexpressing lines and mutants were used in subsequent experiments.

### *TGase* enhanced CO_2_ assimilation in tomato plants

**Fig. 1:**
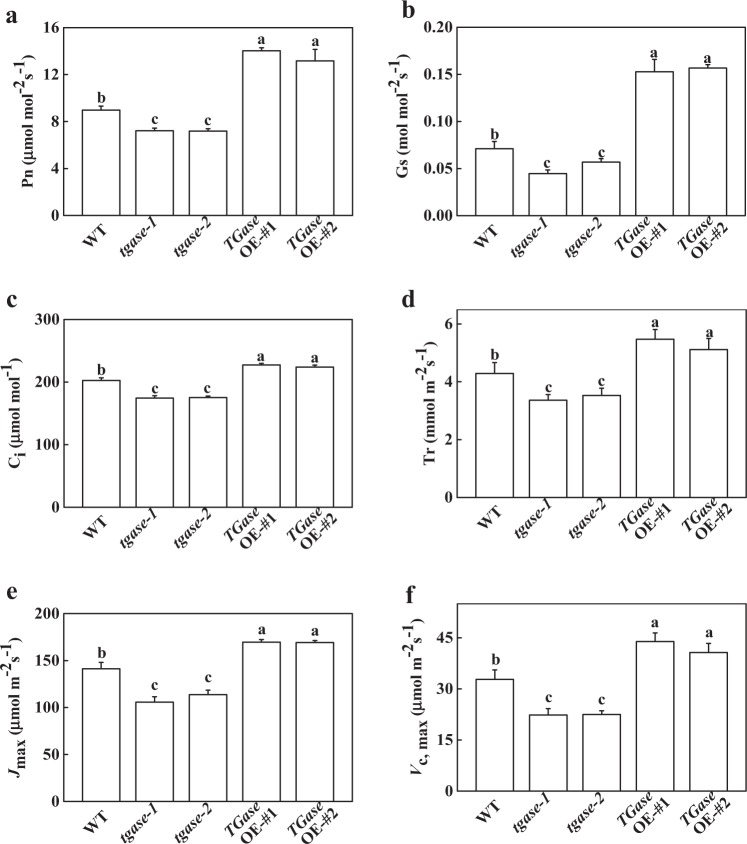
Effects of *TGase* overexpression (OE-#1 and OE-#2) and mutation (*tgase-1* and *tgase-2*) on photosynthesis. Net photosynthetic rate (Pn) (**a**), stomatal conductance (Gs) (**b**), intercellular CO_2_ concentration (Ci) (**c**), transpiration rate (Tr) (**d**), maximum ribulose-1,5-bisphosphate (RuBP) regeneration rate (*J*_max_) (**e**), and maximum ribulose-1,5-bisphosphate carboxylase/oxygenase (Rubisco) carboxylation rate (*V*_c,max_) (**f**). Each histogram represents a mean ± SE of three independent experiments (*n* = 3). Different letters indicate significant differences between treatments (*P* < 0.05) according to Duncan’s multiple range test

TGases are closely related to photosynthesis in plants. To reveal the role of TGase in photosynthesis capability, we first analyzed the differences in the parameters of gas exchange among the WT, *tgase-1*, *tgase-2*, OE-#1, and OE-#2 plants. The results indicated that deficiency of *TGase* significantly inhibited net Pns compared to WT (Fig. 1a). Moreover, other gas exchange parameters, such as stomatal conductance (Gs), C_i_, and transcription rate (Tr), were also reduced in the *tgase* mutants (Fig. 1b–d). In contrast, the Pn, Gs, Ci, and Tr increased in the OE-#1 and OE-#2 lines compared with WT plants (Fig. 1a–d). To further examine the possible mechanism of TGase regulation of photosynthesis, we used Farquhar’s model to analyze the *V*_c,max_ and the *J*_max_. The results showed that *TGase* deficiency decreased *V*_c,max_ and *J*_max_; however, compared with those in WT, *V*_c,max_, and *J*_max_ increased in the *TGase*OE plants (Fig. 1e, f). Thus, TGase-mediated CO_2_ assimilation and RuBP carboxylation and regeneration capabilities in tomato plants.

### TGase promoted the gene expression and activity of the Benson–Calvin cycle

**Fig. 2:**
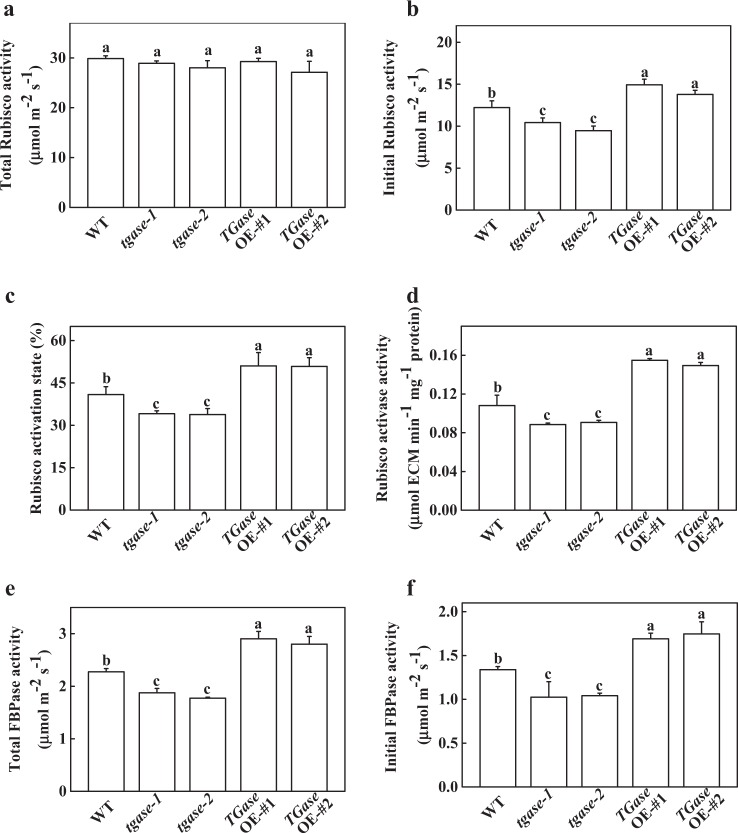
Effects of *TGase* gene overexpression (OE-#1 and OE-#2) and mutation (*tgase-1* and *tgase-2*) on the total and initial carboxylation activity of Rubisco (**a**, **b**), the activation status of Rubisco (**c**), the activity of Rubisco activase (**d**), and total and initial activity of fructose-1,6-bisphosphatase (FBPase) (**e**, **f**). Each histogram represents a mean ± SE of three independent experiments (*n* = 3). Different letters indicate significant differences between treatments (*P* < 0.05) according to Duncan’s multiple range test

To understand the possible role of TGase in regulating the capability of photosynthesis, we further analyzed the activity of FBPase, which plays key roles in RuBP regeneration. The results showed that changes in the endogenous TGase activity did not induce a significant change in total Rubisco activity (Fig. 2a), but the initial Rubisco activity showed significantly differed among the different genotypes, resulting in a decreased and increased Rubisco activation state in the *tgase* mutants and *TGase*OE plants, respectively (Fig. 2b, c). Similarly, the activity of RCA was reduced in *tgase* mutants but elevated in *TGase*OE plants (Fig. 2d). Moreover, *tgase-1* and *tgase-2* presented decreased the total FBPase activity, while high FBPase activity was detected in the *TGase*OE plants (Fig. 2e). Furthermore, there was a similar change in the initial FBPase activity in the *TGase*OE plant (Fig. 2f).

**Fig. 3:**
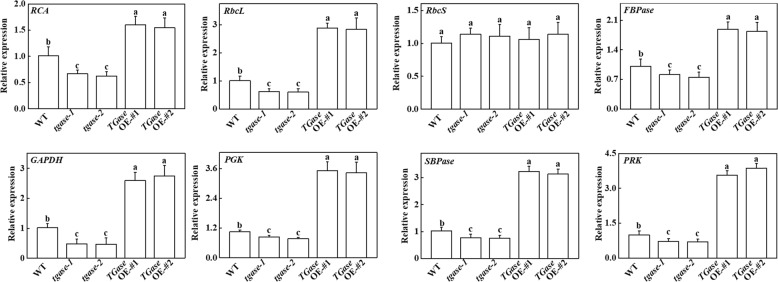
Changes in the expression of Benson–Calvin cycle-related genes in overexpression (OE-#1 and OE-#2) and mutant (*tgase-1* and *tgase-2*) plants. Each histogram represents a mean ± SE of three independent experiments (*n* = 3). Different letters indicate significant differences between treatments (*P* < 0.05) according to Duncan’s multiple range test

**Fig. 4:**
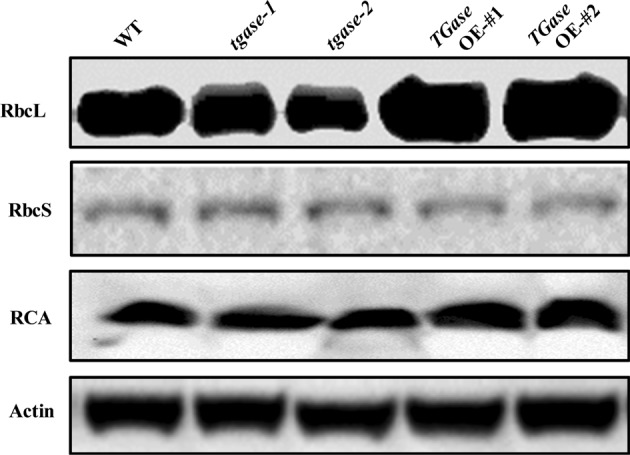
Effects of *TGase* gene overexpression (OE-#1 and OE-#2) and mutation (*tgase-1* and *tgase-2*) on the protein levels of RuBisCO larger subunit (RbcL), RuBisCO small subunit (RbcS), and RuBisCO activase (RCA)

To further analyze the function of TGase in the regulation of photosynthesis, we examined the transcripts of genes related to the Benson–Calvin cycle, including *RCA*, *RbcL*, *RbcS*, glyceraldehyde-3-phosphate dehydrogenase, *FBPase*, sedoheptulose-1,7-bis-phosphatase, ribulose-5-phosphate kinase, and glycerate-3-phosphate kinase. Except for the expression level of the *rbcS* gene, the expression levels of the other genes were upregulated in *TGase*OE plants (Fig. 3). In contrast, deficiency of the *TGase* gene led to downregulation of these genes (Fig. 3). Moreover, the protein levels of rbcL, rbcs, and RCA presented similar results as those of their transcription levels. The results showed that *TGase* deficiency and overexpression induced decreases and increases in rbcL and RCA contents, respectively (Figs. 4 and S5), which are consistent with the total Rubisco activity.

### *TGase* induced a reduced redox status and activated antioxidant enzymes

**Fig. 5:**
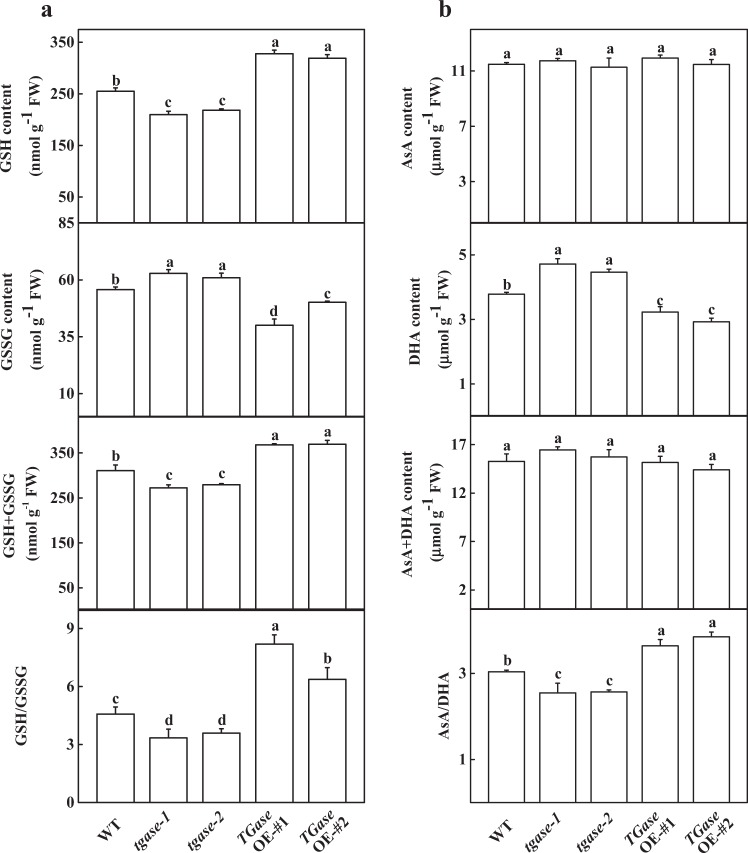
Effects of *TGase* overexpression (OE-#1 and OE-#2) and mutation (*tgase-1* and *tgase-2*) on the glutathione content (**a**) and ascorbate content (**b**). Each histogram represents a mean ± SE of three independent experiments (*n* = 3). Different letters indicate significant differences between treatments (*P* < 0.05) according to Duncan’s multiple range test

It is well known that redox posttranslational modifications can regulate Calvin cycle enzymes. To reveal the mechanism by which TGase mediates Rubisco and RCA activity, we further examined the different status of glutathione and ascorbate contents. The GSH content was decreased in *tgase* mutants, whereas high TGase activity in the *TGase*OE plants resulted in GSH content accumulation compared to WT (Fig. 5a). However, *TGase* deficiency led to an increase in GSSG content but a decrease in the *TGase*OE plants compared to WT (Fig. 5a). The changes in total glutathione (GSH + GSSG) were similar to those of GSH (Fig. 5a). Importantly, the ratio of GSH/GSSG showed a decline in the *tgase* mutants, whereas it increased in the *TGase*OE plants (Fig. 5a). The content of AsA was not affected by different TGase activity levels in plants (Fig. 5b). *TGase* deficiency led to an increase in the oxidized ascorbate (DHA) content but a significant decrease in AsA in the *TGase*OE lines (Fig. 5b). Although the total ascorbate (AsA + DHA) content was not influenced by the modulation of TGase activity, the AsA/DHA ratio significantly decreased and increased in the *tgase* mutants and overexpression plants, respectively (Fig. 5b).

**Fig. 6:**
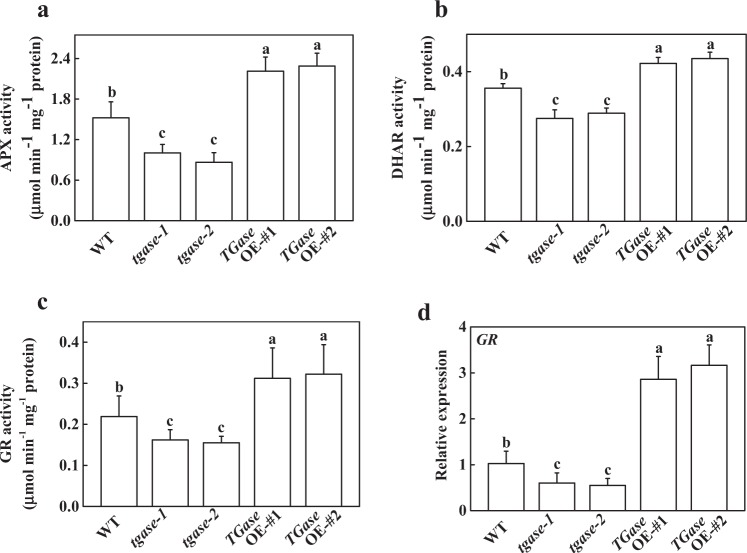
Effects of *TGase* overexpression (OE-#1 and OE-#2) and mutation (*tgase-1* and *tgase-2*) on the activity of ascorbate peroxidase (APX) (**a**), dehydroascorbate reductase (DHAR) (**b**), and glutathione reductase (GR) (**c**), and on the expression of *GR* (**d**). Each histogram represents a mean ± SE of three independent experiments (*n* = 3). Different letters indicate significant differences between treatments (*P* < 0.05) according to Duncan’s multiple range test

TGase can maintain cellular redox status by mediating the enzymatic activity of the AsA–GSH cycle. In this study, APX, DHAR, and GR, which are involved in the AsA–GSH cycle, were analyzed. The results showed that those activities were inhibited in the *tgase* mutants but were induced in the *TGase*OE plants (Fig. 6a–c). Moreover, the expression of *GR* was consistent with its activity (Fig. 6c, d).

## Discussion

TGases are widely distributed in plants and have been found to be related to all stages of plant growth, programmed cell death, senescence, and stress^37^. However, until now, there has been no suitable transgenic plant with decreased TGase activity. Moreover, the best tobacco and *Arabidopsis* experimental lines overexpressed the plastidial transglutaminase from maize, but researchers have not analyzed whether the photosynthetic capability changes in the overexpression plants resulted from the alteration of the Calvin cycle and the promotion of carbohydrate metabolism^21,38,39^. In this study, we explored the function of *TGase* using a reverse genetic strategy. We found that high endogenous TGase activity is involved in the enhancement of photosynthesis in *TGase*OE plants, but *TGase* deficiency led to decreased photosynthetic capability (Fig. 1), which suggested that TGase may play crucial roles in the regulation of photosynthesis. The reduction in the rate of photosynthesis in the *tgase* mutants was associated with a decrease in Gs and Ci (Fig. 1). Our previous study suggested that the application of 8 mM Put enhances stomatal opening and promotes photosynthesis in cucumber^40^. Here, we also observed increased Ci in the *TGase*OE plants but not in the mutants. These results might be due to the positive effect of TGase on the PA level change, which resulted in a significant positive correlation between PAs and the Pn.

To date, several studies have reported that TGase regulates the thylakoid structure and LHCII^22,41^. For example, overexpression of the maize *TGase* could remodel tobacco thylakoids^38,42^. However, little is known about whether TGase can regulate the Calvin cycle and its associated processes. Hence, in this study, we used the A/Ci curve and the Rubisco and FBPase activities to show some evidence for TGase mediation of photosynthetic capability due to the positive regulation of Calvin cycle enzyme activity. We observed that Calvin cycle-related genes were downregulated in the *tgase* mutant (Fig. 3). Furthermore, TGase deficiency also led to a significant decline in RCA activity and initial Rubisco and FBPase activity, as well as a reduction in *V*_c,max_ and activity of RCA, with almost no change in the total activity of Rubisco (Figs. 2 and 4). In contrast, overexpression of *TGase* increased the expression and activity of these genes (Figs. 2 and 3), indicating that TGase mainly regulated the action of Rubisco activities to activate the state of Rubisco. *rbcS* and *rbcL* encode small and large Rubisco subunits, respectively, which compose the Rubisco holoenzyme and are tightly coordinated, but the transcripts of *rbcS* and *rbcL* are not correlated^43^. Indeed, although TGase positively mediates the transcription of *rbcL*, it did not mediate the expression level of *rbcS* (Fig. 3b, c). This phenomenon may be due to the synthesis of rbcS being different because a study showed that overexpression of *rbcS* did not lead to an increase in the content of Rubisco^44^. Alternatively, pools of cellular glutathione could be a repressor of rbcL proteins when glutathione is in the oxidized state^45^. Considering that the redox status of glutathione was regulated by TGase, it is possible that TGase affects the levels of *rbcL* (Fig. 5). The regeneration of RuBP is involved in not only the electron transport chain of photosynthesis but also the Rubisco enzyme in the Calvin cycle^46^. Both the expression and initial activity of FBPase were reduced in the *tgase* mutant plants (Fig. 2f). The lack of activation of RCA or FBPase was often related to a decline in *J*_max_ (Fig. 1e), and cellular glutathione homeostasis is related to the activities of these enzyme^47^.

Some reports have shown that GSH plays a key role in Rubisco activation by enhancing thiol and disulfide exchanges^48^. The high ratio of GSH to GSSG increased the activation level of RuBisCO and consequently CO_2_ assimilation^49^. In addition, the glutathione redox could regulate translation of RbcL through modulating the ROS in the chloroplast^50^. Overexpression of *S-adenosylmethionine synthetase* 1 (a PA biosynthesis gene) significantly increased the AsA/DHA and GSH/GSSG ratios in tomato^48,51,52^. The mechanism of TGase positively regulates the activation state of Rubisco, which may be involved in glutathione redox status changes. Interestingly, in the present study, high ratios of GSH/GSSG and AsA/DHA were observed in *TGase*OE lines (Fig. 5), which was caused by an increase in MDAR and GR enzymatic activity (Fig. 6). Activation of the GSH–AsA cycle in *TGase*OE plants could maintain a reducing state of the chloroplast, which helps to activate Rubisco. In addition, TGase could bind to the large subunit of ribulose bisphosphate carboxylase–oxygenase, thereby directly activating the activity of Rubisco^16^. This phenomenon is in agreement with the results by which high TGase activity promoted RCA activity and protein content in *TGase*OE plants (Figs. 2d and 4). On the other hand, maintaining the high AsA/DHA and GSH/GSSG ratios may be involved in hormone signaling, cell division, and other physiological processes^49^.

It is well known that maintaining cellular redox status requires a turnover rate of the ascorbate–glutathione cycle, and APX activity may have an important role in ROS scavenging^53^. Here, we found that a deficiency in *TGase* resulted in a decreased activity of antioxidant-related enzymes, while overexpression of *TGase* increased their activities, as observed in the overexpression of maize plastidial TGase in tobacco^42^. These results indicated that high endogenous TGase activity induced antioxidant enzymes to regulate the redox system of the plants. This finding further revealed the cross-link between TGase and the glutathione system^42^.

In conclusion, the current study indicated that manipulation of endogenous TGase activity by overexpression of *TGase* could promote the CO_2_ assimilation rate through activating the Calvin cycle enzymes. Moreover, TGase-inducible changes in cellular redox homeostasis may be involved in activation of Calvin cycle enzymes.

## Supplementary information

Supplementary Information
