# Supplementary material for: TGase positively regulates photosynthesis via activation of Calvin cycle enzymes in tomato
Source: Hortic Res. 2019 Aug 1;6:92. doi: 10.1038/s41438-019-0173-z (PMC6804539; doi:10.1038/s41438-019-0173-z)
Supplement: Supplementary file 1 — Supplementary Information [file 41438_2019_173_MOESM1_ESM.docx]

**Fig. S1** Expression of *TGase* in different organs of tomato by quantitative real-time PCR (qPCR). Each histogram represents a mean±SE of three independent experiments (n=3). Different letters indicate significant differences between treatments (*P*<0.05) according to Duncan’s multiple range test.

**Fig. S2** Relative transcription level of *TGase* in each overexpression line. Each histogram represents a mean±SE of three independent experiments (n=3). Different letters indicate significant differences between treatments (*P*<0.05) according to Duncan’s multiple range test.

**Fig. S3** Identification of *tgase* mutants. a, DNA sequence comparison of WT, *tgase-1* and *tgase-2*. Sequencing analysis showed that *tgase-1* mutant contained the 10 bp deletion in the exon and *tgase-2* mutant contained a 1 bp insertion in the exon. b, Sequencing result of *tgase-1* mutant. c, Sequencing result of *tgase-2* mutant.

**Fig. S4** The activity of TGase in WT, *tgase-1*, *tgase-2* and *TGase*OE plants. Each histogram represents a mean±SE of three independent experiments (n=3). Different letters indicate significant differences between treatments (*P*<0.05) according to Duncan’s multiple range test.

**Fig. S5**. The relative intensity of protein changes in WT, *tgase-1*, *tgase-2* and *TGase*OE plants. Each histogram represents a mean±SE of three independent experiments (n=3). Different letters indicate significant differences between treatments (*P*<0.05) according to Duncan’s multiple range test.

**Table S1.** The primer pairs used in this study.

| Primer name | Sequence (5’–3’) | Purpose |
| --- | --- | --- |
| TGase-F | TTGGCGCGCCATGGTTGCTCGGAGACTCGCCGTTA | TGase-HA vector construction |
| TGase-R | CGGGGTACCACTGCTACCTGCAAAGAGGTCAATG |  |
| gRNA target sequences | GGCCCTTCAGTCTCATTACC | gRNA target sequences |
| CRISPR1-F | CGGCGAAACATCATTTCAGA | *tgase* mutants identification |
| CRISPR1-R | AGGAAGCTCCTCCCCACTAG |  |
| *RCA*-F | CTGTTGGTCATCCGATGTGT | qPCR |
| *RCA*-R | CCCAAGGTTTCAAACAGGAA |  |
| *rbcL*-F | ACCGCAAATACTACCTTGGC | qPCR |
| *rbcL*-R | CCACCAGACATACGTAACGC |  |
| *rbcS*-F | TTGCTTGGAATTCGAGACTG | qPCR |
| *rbcS*-R | CTCTTGAACCTCAGCCAACA |  |
| *FBPase*-F | GAAGAGAAATGGCATCAGCA | qPCR |
| *FBPase*-R | AGTGAGTCCAGAAGGATGGG |  |
| *GAPDH*-F | GATTGGAGAGGTGGAAGAGC | qPCR |
| *GAPDH*-R | ACCACGGACACATCAACAGT |  |
| *PGK*-F | GAAGGGCAAGAAAGTCTTCG | qPCR |
| *PGK*-R | AGTGTTTGATGGTAGGGATGG |  |
| *SBPase*-F | AGTTGGTGCTGCTGTTTGAG | qPCR |
| *SBPase*-R | TTCGCGATGCTCTAGAAAGA |  |
| *PRK*-F | GGGATATGGCTGAAAGAGGA | qPCR |
| *PRK*-R | CTGAGTTGGGAGCACTTCAA |  |
| *TGase*-F | ATTGGAAGAACGGGACAGGT | qPCR |
| *TGase*-R | TGCATTTGCGAACTGGACAA |  |
| *actin*-F | TGGTCGGAATGGGACAGAAG | qPCR |
| *actin*-R | CTCAGTCAGGAGAACAGGGT |  |
